# Gene Editing of Microalgae: Scientific Progress and Regulatory Challenges in Europe

**DOI:** 10.3390/biology7010021

**Published:** 2018-03-06

**Authors:** Andrew Spicer, Attila Molnar

**Affiliations:** 1 Algenuity, Eden Laboratory, Bedfordshire MK43 9ND, UK; 2 Institute of Molecular Plant Sciences, University of Edinburgh, Edinburgh EH9 3BF, UK; amolnar@exceed.ed.ac.uk

**Keywords:** CRISPR, transgenic, GMO, GMM, gene editing, Cpf1, DSB, NPBT, SDN

## Abstract

It is abundantly clear that the development of gene editing technologies, represents a potentially powerful force for good with regard to human and animal health and addressing the challenges we continue to face in a growing global population. This now includes the development of approaches to modify microalgal strains for potential improvements in productivity, robustness, harvestability, processability, nutritional composition, and application. The rapid emergence and ongoing developments in this area demand a timely review and revision of the current definitions and regulations around genetically modified organisms (GMOs), particularly within Europe. Current practices within the EU provide exemptions from the GMO directives for organisms, including crop plants and micro-organisms that are produced through chemical or UV/radiation mutagenesis. However, organisms generated through gene editing, including microalgae, where only genetic changes in native genes are made, remain currently under the GMO umbrella; they are, as such, excluded from practical and commercial opportunities in the EU. In this review, we will review the advances that are being made in the area of gene editing in microalgae and the impact of regulation on commercial advances in this area with consideration to the current regulatory framework as it relates to GMOs including GM microalgae in Europe.

## 1. Introduction

Most of the food or feed that has been consumed by humans, cattle, and pets comes from plants, animals, fungi, and even microorganisms grown, bred, and improved by mankind over thousands of years. Through the centuries, by using different breeding methods, we have managed to select the best-performing individuals and strains of crop plants, animals, fungi, etc., with enhanced growth rates and productivities, and improved nutritional composition and health.

As a general rule, the improved organisms arose from naturally occurring variations in the genetic make-up of those plants, animals, fungi, etc., or from natural partial or full genome duplications or breeding (crosses) between closely related organisms. For the past 90 years, radiation-induced changes in genomes [1] have been used as a way to accelerate the development of improved organisms for exploitation by man. Since the 1970s, it has also become possible to modify the genetic make-up of living cells and organisms using biotechnology-based techniques, usually called genetic engineering. In general, genetically engineered crops or animals are produced through modification of the genetic material by introduction of a specific piece of DNA called a transgene (generally considered to be a gene derived from a different, unrelated species, such as a bacterial gene introduced into a higher plant, or even a synthetic gene) to give the organism a new property (e.g., resistance to a disease or a specific herbicide, improvement of food quality, or increased crop productivity).

Organisms produced in this way are called “genetically modified organisms” (abbreviated commonly as GMOs). It follows that food and feed that contain or consist of such GMOs, or are produced from GMOs, would be “genetically modified (GM) food or feed”.

The development of modern molecular approaches to genetically improving organisms is necessitating a reconsideration of the definition and risk assessment process associated with the current GMO regulatory framework in Europe but also globally. In this review we will consider the current regulatory framework within which gene-edited algae still fall and also summarise and highlight advances in technology development with recommendations on steps that should be taken.

## 2. Definitions of GMOs and GMMs and Exemptions in Europe

European Union (EU) legislation (Article 2 of EU Directive 2001/18/EC) [2] defines a genetically modified organism (GMO) as “an organism, with the exception of human beings, in which the genetic material has been altered in a way that does not occur naturally by mating and/or natural recombination.” Genetically Modified Microorganisms are often referred to as GMMs. Directive 2009/41/EC [3] defines microorganisms as “any microbiological entity, cellular or non-cellular, capable of replication or of transferring genetic material, including viruses, viroids, animal and plant cells in culture”. This would include all microalgae, encompassing both eukaryotic and prokaryotic (cyanobacteria) microalgae.

As such, under this broad definition, many experimentally produced organisms fall under this umbrella. This includes the following organisms:

1. All organisms where a ‘transgene’ or more than one transgene encoded by DNA has been integrated stably into the genome (nuclear, mitochondrial, or other genome such as chloroplast) such that it is inherited by offspring or daughter cells—this includes cisgenes (gene is from the same species or a closely related species) and transgenes (a foreign gene from a completely unrelated species or a synthetic, artificially designed gene for a specific function). For the sake of this paper, the broad term ‘transgene’ will be inclusive for the above genes (i.e., cis and transgenes);
2. All organisms where an artificial chromosome has been introduced into the organism such that it is replicated alongside the natural chromosomes and stably inherited in offspring and/or daughter cells;
3. All organisms where a native gene has been stably mutated by non-natural, laboratory means—including the introduction of nucleotide substitutions, deletions, rearrangements, duplications, insertions, or inactivation by any one of the following methods:
  - UV- or radiation-induced mutagenesis;
  - Chemical mutagenesis;
  - Gene editing by site-directed nucleases including Clustered Regularly Interspaced Short Palindromic Repeats/CRISPR associated protein (CRISPR/Cas), Transcription Activator-Like Effector Nucleases TALENs), and Zinc Finger Nucleases (ZFNs)
  - Site-specific recombinases, integrases, or meganucleases.

It might be surprising to the average reader to learn that organisms derived from chemical or UV/radiation mutagenesis in a laboratory would be broadly included under the GMO umbrella in Europe. In practice, however, organisms produced through chemical or UV/radiation mutagenesis are considered to be excluded or given an exemption with regard to regulations that consider the risk assessments that need to be undertaken for controlled release or utilisation of such organisms. This exemption is made as organisms produced in this way generally have a long-standing safety record. Random mutation occurs spontaneously in nature and is the basis for the genetic variation we see within species. The laboratory-driven mutagenesis techniques of UV/radiation and chemical mutagenesis do not result in the introduction of any new genetic material into the organism. These methods effectively accelerate what would happen in nature over years or centuries; they create mutants, but no foreign gene is added. This is an important distinction that we will come back to with regard to a similar consideration that should be applied to gene-edited organisms where no foreign gene has been added to the genome.

## 3. Risk Assessments: Relative Risks are Key

The risk assessment debate is most developed around and within applications of GMOs in food and feed but specific regulations and risk assessment requirements also apply to application within other sectors including areas such as fuels, cosmetics, personal care, and household products. The European Food Standards Agency (EFSA) states that identification, characterisation, and handling of risk(s) associated with GMOs should follow a structured approach, consisting of three interconnected elements: risk assessment, risk management, and risk communication [4]. All three elements need to be considered and documented. Risk assessment is a scientific exercise. Principles for risk assessment of GMOs are described in Directive 2001/18, Annex II, supplemented by Commission Decision 2002/623/EC [2,5]. This is related to production or application of GMOs in a ‘deliberate release’ scenario such as outdoor cultivation or sale of a GMO or GMO-derived product for human or animal consumption. The alternative to ‘deliberate release’ is contained use. The GMO Regulations define contained use as 'any activity in which organisms are genetically modified or in which such organisms are cultured, stored, transported, destroyed, disposed of or used in any other way and for which specific containment and other protective measures are used to limit their contact with the general public and the environment’. The contained use of genetically modified microorganisms is regulated by Directive 90/219/EEC [6], as amended by Directive 98/81/EC [7]. Most of the controversy and debate around the production and use of GMOs is limited to risk assessments associated with deliberate release.

An extensive overview of risk assessment procedures is provided by the Scientific Committee of the EFSA [8]. The risk assessment involves generating, collecting, and assessing information on a given GMO in order to determine its potential impact on human and/or animal health and the environment compared with the nonmodified organism from which it is derived. To carry out the risk assessment, sufficient scientific and technical data must be available to arrive at qualitative and/or quantitative risk estimates. In general, a risk assessment includes a consideration of the parental organism including its associated biology, likelihood of spread in the environment and any barriers that may act to limit this spread, the nature of the genetic modification, and the presence of the GMO or GMM and its derivatives, including DNA, in the final food or feed product. GMMs used for food and feed purposes can be differentiated on the basis of their use in (i) GMOs deliberately released into the environment, according to Directive 2001/18/EC [2], and used as food or feed or contained in food or feed; (ii) GMOs deliberately released into the environment, according to Directive 2001/18/EC [2], and used for the production of food or feed; (iii) GMOs used for the production of food or feed under ‘contained use’.

For uses under (i) and (ii), a full risk assessment according to Regulation 1829/2003 [9] in combination with Directive 2001/18/EC is required and is covered by this guidance. With regard to uses as in (iii), i.e., GMOs used for food or feed production under containment, this guidance covers the assessment of the final product to be used as food or feed for placing in the market, while taking into account the characteristics of the GMO, but does not cover the production process as performed under containment. Regulation 1829/2003 specifically covers traceability of the GMO including the methods to be used. While most methods rely upon detection of the GMO using nucleic-acid-based approaches, some consider detection of phenotypic traits as well as protein [10].

Risk assessments associated with GMOs are related primarily to the risk of damage that the modified organism may produce to the environment, humans, or animals, including the risk of the ‘transgene’ spreading by gene transfer to other organisms or the risk of the GMO itself spreading as an aggressive or invasive organism outside of its primary site of production. Indeed, most of the risk assessment process considers the relative harm that could be caused either by the altered organism retaining a new undesirable or hazardous property that is attributed to the modified DNA or the ‘transgenic’ DNA spreading to other organisms including microorganisms, higher plants, or even animals. Notable examples of transgenes where significant risk could be considered are transgenes encoding herbicide resistance, antibiotic resistance, or enhanced growth under natural or agricultural field conditions. 

Mutations produced by chemical or UV/radiation mutagenesis are random and often a cell or organism produced in this way contains multiple independent mutations at different sites within the genome. Due to the random nature of these mutations, it could be considered that any or all of these mutations could occur naturally; they are not specifically engineered—they are organisms (mutants) where mutagenesis (called classical or random mutagenesis since very long ago) has allowed the selection of desirable phenotypes by the breeder. As such, there is no transgene that would be the subject of a risk assessment, although one could argue that any of the mutated native genes could be associated with a potential or hypothetical—even if not directly measurable—risk with regard to acquisition of a new trait, including altered growth properties or relative resistance to a specific herbicide.

A comparison of the relevant methods for genetic modification and the associated risk assessments and exemption status is summarised in Table 1.

## 4. Gene Editing: What Is It? Why Is It So Controversial?

Gene editing refers to a group of techniques where specific genetic modifications are designed into the native genome of a given organism by application of one of a suite of approaches (see below) that permit the creation of a cell or entire organism with precise genetic modifications in native gene sequences without the need to integrate transgene DNA into the genome. The gene editing approaches considered herein rely upon directing a molecular tool to a specific selected site in the genome to create a double-strand break (DSB) in the DNA sequence. The cell re-joins cut DNA predominantly without reference to the original DNA sequence. This mechanism is error prone and can result in random mutations (nucleotide insertions or deletions) at the site of DNA repair. If the targeted DNA is within a gene that encodes a protein, this can generate mutations that prevent the production of a functional protein. Although at much lower frequency, precise DNA editing can also be induced if DNA is provided as a repair template via the homology-directed DNA repair pathways. Thus, the gene editing technology is ideal for producing novel genetic variants in a sequence-specific manner.

All of these methods are grouped under a common set of approaches referred to as Site-Directed Nucleases (SDN) because they rely upon directing a specific DSB in the DNA, created by a nuclease, to a desired site within a complex genome. Sometimes, these methods are collectively called New Plant Breeding Techniques (NPBT) [13] when used to refer to crop plants, or Oligonucleotide Directed Nucleases (ODNs) when used in a more general context of selective mutagenesis, as compared with spontaneous or induced random mutagenesis—the main current technology for cultivar or strain improvements.

The first approach that gained attention with regard to established gene editing was the application of protein domains called zinc fingers (DNA binding proteins) fused to nucleases [14] (DNA cleavage proteins), creating zinc finger nucleases or ZFNs. The zinc finger domains direct pairs of proteins to a specific DNA sequence, with the protein complex assembling at the desired site in the genome to effectively form a pair of molecular scissors to create a DSB at that site. The next approach that increased specificity and precision of the DSB process was the discovery and application of proteins referred to as TALEs (Transcription Activator-Like Effectors) [15] fused to nuclease domains to create so-called TALENs, again to direct a set of molecular scissors to a specific genomic target sequence to create a precise genome change at that site. TALENs increased the capacity and opportunities to apply gene editing across diverse platforms including within commercial organisations, in the clinic, and in domesticated/herd animals [16].

The true step-change in gene editing arose, however, through the discovery of [17] and ultimate application of the CRISPR/Cas [18] system, in which a common endonuclease (Cas9 most usually, but other similarly acting proteins such as Cpf1 (also known as Cas12) have now been identified and successfully applied) is directed to a desired genomic DNA target through the use of a short, easy-to-engineer RNA sequence referred to as a guide sequence. CRISPR/Cas has made gene editing technology readily accessible to clinicians, researchers, and breeders. Indeed, it has already been applied successfully within a molecular medicine context in the clinic in humans [19,20,21], and modified fungi [22] and plants [23] created using CRISPR/Cas have now been approved for production for human consumption [24]. The advent of these advanced genome modification technologies has created a stir of activity globally within regulatory bodies that work within the various economies of the world to assess, rule, and oversee safe application of technology, particularly ruling on those technologies that introduce products into the human or animal food chain. This includes organisations such as EFSA, the US Department of Agriculture’s Animal and Plant Health Inspection Service (APHIS), the German Federal Office of Consumer Protection, and even the European Court of Justice. Indeed, a proposed regulatory framework has been put forward to consider the relative risk assessments that should be or could be applied to crops or organisms produced through gene editing [11]. Furthermore, the Norwegian Biotechnology Advisory Board has made recommendations along the lines that a new three-tier system could be envisaged where reporting, risk assessment, and regulation are applied at increasing levels [25].

Because of the subtle and specific nature of the mutations that can be created and the fact that a foreign transgene does not need to be integrated into the genome and is, therefore, not stably inherited, it has been strongly argued that such organisms produced by gene editing including plants, fungi, animals, and microorganisms should not be considered under the current broad umbrella definition of GMO [13,24]. Indeed, the ability to distinguish if an organism has been produced through gene editing or is, in fact, a naturally occurring variant organism carrying a desired mutation, or one produced by conventional mutagenesis methods, is essentially impossible given any technology. This goes against EU legislation that requires that GMOs be identifiable and traceable using suitable detection methods [9,10]: this directive stems from the assumption that was made when the GMO definition was originally put into action in the early 2000s, when it was widely assumed that all GMOs would result from integration of a transgene into a genome—a transgene that could then be detected using available detection methods.

In short, we believe that the current EU definition of GMO and the directives associated around risk and relative safety of organisms produced through conventional transgenesis cannot be applied in the same way to organisms generated through gene editing where a modification in a native gene is the only introduced modification. We also believe that there is no rational reason why an organism created in this manner would be considered firmly under the GMO umbrella while an organism made by chemical or UV/radiation mutagenesis would be considered exempt from the GMO umbrella and its associated regulations. Indeed, all such variants are not fundamentally different from the spontaneous mutations that arise in nature in crop plants and animals all the time and have been the basis for traditional crop and animal selection and improvements. 

In many regards, gene editing could be seen as genetic engineering taken to the height of its possible precision. Indeed, using the CRISPR/Cas system, human genetic disease is already being treated in the clinic [24]. It is perhaps ironic then that while this technology has been approved for application in humans for molecular medicine [24], plants, fungi, or microorganisms created using the same level of precision and the same approach have, for the most part, not been ruled upon within Europe with regard to their use in food and feed production. Specifically, while some national bodies [26] have made recommendations that SDN-modified organisms where no foreign transgene has been integrated into the genome should not be considered GMOs, the EU has not made any specific ruling (see below). This includes an official EU position on any organism including micro or macroalgae or even bacteria (including cyanobacteria) produced through gene editing approaches. Recently, however, a formal opinion from an advocate general in the European Court of Justice suggests that crops and drugs created using powerful gene editing techniques such as CRISPR/Cas9 might not need to be regulated by the strict European Union rules that govern genetically modified organisms (GMOs) [27].

## 5. Gene Editing in Algae: Current State of the Art

Genetic modification techniques in algae are largely restricted to laboratory exercises and not to algae produced commercially for GMO-derived products sold in today’s market. Genetically modified algae mostly include a handful of strains, with most studies carried out on the laboratory model green microalga, *Chlamydomonas reinhardtii*. Overall, approximately 20 different microalgal species including cyanobacteria have been successfully genetically modified to date. A smaller number of macroalgae have also been genetically modified [28]. In the vast majority of cases, these modifications would fall under the broad and classical GMO view where a transgene has been integrated into the nuclear or, in some cases, the chloroplast genome of the given algal species. As such, all these micro and macroalgae would fall broadly under the GMO umbrella. There has been some debate as to whether or not GM algae fall under Genetically Modified Microorganisms (GMMs) or Genetically Modified Plants, or even both categories, and questions as to how this might impact any risk assessments that would need to be performed. EFSA reviewed this area and commented as follows: “According to our experience, the current European legislative framework for GMOs and for food/feed products of microbial origin (whether they are GM or not) would cover GM microalgae sufficiently.” Regardless of the category of organism, the regulations considered herein relate to GMOs broadly and would include both GMMs and GM Plants. With regard to production of GM algae beyond labscale, contained use—often double-contained or restricted-access greenhouse—is considered to be a safe working model at the current time, although outdoor trials have gone ahead and have been reported in the USA [29].

**Figure 1:**
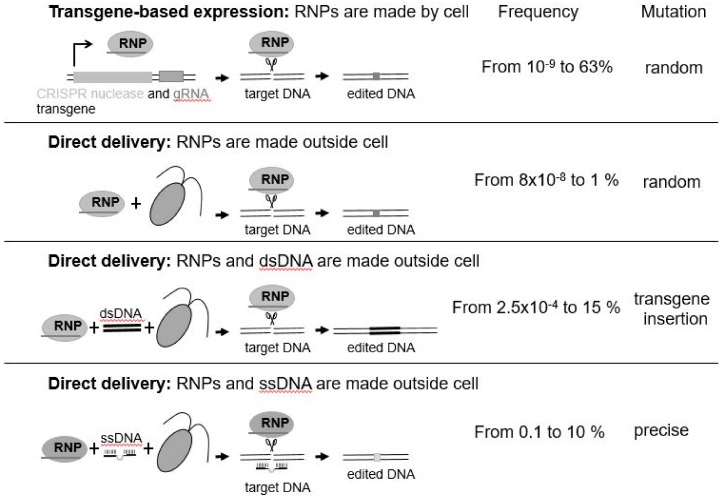
Gene editing approaches in microalgae. Relative frequency range of successful mutations reported from each approach are provided. RNP = ribonucleoprotein; gRNA = guideRNA; dsDNA = double-stranded DNA; ssDNA = single-stranded DNA. Transgene-based expression [31,32,33,38]; Direct delivery, RNPs made outside cell [34,35]; Direct delivery, RNPs and dsDNA made outside cell [36]; Direct delivery, RNPs and ssDNA made outside cell [37].

It is only very recently that any progress has been made with gene editing in algae. The first reported successful application of a gene editing approach in a microalga was achieved in the marine diatom, *Phaeodactylum tricornutum*, by a team at Cellectis in France using TALENs [30]. More recently, application of CRISPR/Cas9 and CRISPR/Cpf1 have been successfully reported in *Phaeodactylum tricornutum* [31], *Thalassiosira pseudonana* [32], *Chlamydomonas reinhardtii* [33,34,35,36,37], and *Nannochloropsis oceanica* [38]. Different methods have been employed to edit the genome of alga species (Figure 1). Some use transgenes as means of stable expression of CRISPR/Cas. Others introduce preassembled active ribonucleoprotein (RNP: enzyme + guideRNA) complexes by electroporation (Figure 1) or take a hybrid approach where Cas9 is produced from a transgene and other editing components are delivered directly to the nucleus [39]. While transgenes may be used to initially deliver the CRISPR reagents, they are not needed once the genome has been edited. Since they are located elsewhere in the genome, transgene-free, genome-engineered progenies can be recovered by mating/breeding. Intriguingly, direct delivery of CRISPR/Cas9 or CRISPR/Cpf1 RNPs completely bypasses transgenesis. Why is this important? It is important as it means that, not only is there no foreign transgene integrated into the resultant algal cell’s genome, but that a foreign transgene was never introduced to effect the desired change—this was, rather, effected by the use of an active protein–RNA complex that was introduced into the cell, and subsequently targeted the desired change to the nuclear genome [35,37]. The half-life of active RNPs is around 24–48 h, greatly reducing any off-target (unwanted) mutations that have already been shown to be very low or virtually nonexistent in plants [40]. Thus, RNPs are considered as safe and efficient molecules to induce the desired mutations without any trade-offs.

The mutations induced by Cas9- or Cpf1-mediated DNA cleavage arise by the cell’s natural process for repairing DNA, which can occur under natural conditions, such as after DNA damage by sunlight. The resulting mutations are random insertions/deletions (Figure 1). In contrast, templated DNA repair—single-stranded DNA (ssDNA) ([37], see Figure 1) and double-stranded DNA (dsDNA)—can result in nucleotide-specific precise DNA replacement. However, bigger insertions such as tags or selection markers may be considered as transgenes depending on the size of integrated DNA.

While it is early days in the application of gene editing approaches to algae, it is likely to become a standard procedure that can be applied to most algal species where DNA, RNA, or protein can be delivered into the cell. This will include microalgae, cyanobacteria, and macroalgae. The power of gene editing lies within the ability to create desired subtle changes to genomes. In microalgae, for instance, inactivation or changes in specific genes that impact the metabolic output including oil composition, protein composition, cell wall composition, motility, nutrient uptake or utilisation, photosynthetic efficiency, and numerous others would be expected to be transformational to the industry. It can be noted that these genome changes would also be achievable through natural or accelerated (that is, induced) mutagenesis and selection for the desired phenotypes if achievable, or by using extensive bioprospecting and genomic sequencing to identify specific underlying genotypes. New gene editing and sequencing technologies greatly facilitate and accelerate the creation of such new variants through such selective mutagenesis, compared with traditional random—and more laborious and uncertain—methods.

The potential positive impact of the application of these strain engineering approaches has been highlighted in the final report of the National Alliance for Advanced Biofuels and Bioproducts (NAABB) consortium, a $48.6 million Department Of Energy (DOE) and private-investor-funded three-year project in the USA [41]. This closeout report concluded that the first primary area of cost reduction in a push towards sustainable and scalable algal commodity products was in the area of new strain development, and that when naturally bioprospected microalgal strains are “combined with genetically modified (GMO) versions of the strain, the cost of algal ‘biocrude’ would be reduced by 85%”. The majority of the genetic targets identified through this body of work were native genes with mutations, therefore made feasible by gene editing. The second area that was identified where significant improvements could be made in the economics of the overall process was in the area of cultivation—specifically, the “development of a new open pond cultivation system, the Aquaculture Raceway Integrated Design (ARID), which uses little energy, extends the growing period, improves productivity, and provides a 16% cost reduction.” Hundreds of millions of dollars and euros have been invested over the past 30 years in an attempt to move microalgal platform technologies towards economic and sustainable scalable models to impact society and the environment as we move towards solving the challenges of an increasing population and reducing resources. For microalgal commodity products, including food and feed but also fuels and fuel additives, low cost, outdoor production, and strain improvements established by approaches including non-GMO directed evolution and gene editing of specific target genes are likely to be key solutions that will be applied in an additive or collective manner.

Unlike niche-focused industries, the algae industry is a global market with broad impact. The algae industry has experienced high levels of competition due to profitability struggles, commodisation, and high exit costs. The emerging algae industry is still, however, an opportunity, with large potential fueled by drivers of increasing population, demand for fuel and food, and the pressing need for sustainable solutions to address climate change. Transparency Market Research [42] valued the 2015 global algae biomass market at $608 million with projections of 7.39% Current Annual Growth Rate (CAGR) to $1.14 billion in 2024 and total algae production at 27,552 tonnes by 2024; the global algal biomass production capacity underpins global markets of $1.38 billion and $616 million for algae oils and proteins, respectively, with CAGRs of 4.3% and 6.27% projected by 2025 and 2022. Despite the current size, growth, and market potential, the algae industry is underachieving and should be hitting market sizes and production levels of at least an order of magnitude higher.

Gene editing approaches will be part of the solution as described above in increasing profitability via improved productivity of strains and improvements in harvestability, processability, and other desirable traits. We caution that the precautionary principle, sometimes heavily applied within regulatory frameworks to novel technologies, can stifle innovation and commercial application if not weighed appropriately against benefits and scientific knowledge.

## 6. Council- and Country-Specific Rulings and Recommendations

What is happening at the moment within and outside of the EU regarding organisms created using gene editing? In the USA, the US Department of Agriculture (USDA) has ruled that the regulations should relate to the product or the organism itself rather than the process. It is tending to rule in favour of gene-edited plants and organisms with regard to approval for growth or entry into the food chain for animals, fungi and plants, or clinical practice for humans. This is the case for a number of other countries outside of Europe, where many are now moving towards a case-by-case consideration process [12]. This is in contrast to the EU and the UK, where current policy and GMO guidelines continue to include plants, animals, fungi, and microorganisms modified by gene editing under the GMO umbrella. It is not that surprising to note, then, that the majority of the commercial and clinical applications or developments of gene editing technologies are happening outside of the EU. This is in spite of the fact that the majority of the initial and even recent innovations around gene editing are credited to groups based in the EU. This includes TALENs and CRISPR/Cas. Thus, economic development in this area is stalled within the EU across all markets and sectors. Companies, in general, are not investing in research and development around this area in the EU [43].

How important is the current EU non-ruling in this area? There are current trade and political talks going on between the USA and EU, the results of which could have a substantial impact on trading relationships with regard to food and feed entering the EU marketplace. Economically, the impact of these rulings should not be understated. Several EU countries have proceeded ahead of any official EU or ECHJ (European Court of Human Justice) decision and have made rulings in favour of a non-GMO label being applied to gene-edited crops. These countries include Sweden and Finland, with the Netherlands and Germany also approaching a decision in favour of a non-GMO label. All these countries that are moving early with regard to making national recommendations have stated that they would defer to EU rulings if and when that is made. One possibility is a total redefinition of the concept of a GMO and the risks and regulations associated therein, such as a definition and risk assessment process that work together on a product or case-by-case basis, considering the pros, cons, and risks, and following a responsible innovation evaluation process [44]. We believe that there is great practical and scientific necessity and a time imperative for moving expeditiously, as both scientific and commercial advances do not allow further hesitation. We would argue strongly along with many others that gene-edited organisms where subtle changes in native genes have been made, where no transgene DNA has been integrated, and particularly where no foreign transgene has been introduced into the cell, should be considered outside the currently restrictive and outdated GMO regulatory umbrella.

## Figures and Tables

**Table 1:** What is a genetically modified organism (GMO)? Types of modifications in broad terms, the nature of the change to the genome, risk management considerations, exemptions that might be granted with regard to classification as GMO or not in the EU, and any rulings of non-GMO from outside Europe are noted.

Type of Modification	Change to Genome	Risk Management	Exemptions Granted (EU)	Ruling of Non-GMO Outside Europe?
Transgene	Stably integrated foreign gene.	Spread of foreign transgene or modified organism in the environment. Risk of harm of transgene product in food chain.	NO	NO
Cisgene	Stably integrated gene: from same species or closely related.	Spread of modified gene or organism in the environment. Risk of harm of cisgene product in food chain.	NO	YES
Artificial chromosome	Stably inherited artificial chromosome.	Spread of artificial chromosome, genes contained therein, or modified organism in the environment due to acquired trait. Risk of harm of transgene or cisgene products in food chain.	NO	NO
Chemical-, UV-, or radiation-induced mutagenesis	Single nucleotide changes, small deletions—usually many/organism—changes in native genes only.	No foreign or artificial gene integrated. Changes only to native DNA but often multiple genetic hits in the genome. Genetic changes can change phenotype including relative growth rate and survivability.	YES	YES
Recombinase- or integrase-driven change	Partial deletions, inversions, duplications, rearrangements or insertions. Recombinase or integrase stably or transiently expressed.	No foreign gene where recombinase or integrase transiently expressed: mods to native genes only. Risk of spread of stably integrated recombinase or integrase. Spread of modified organism.	NO	YES
Gene edit: transgene-driven change (stable or transient)	Subtle mutation to native genes. Stable transgene-driven = conventional transgenic; transient transgene: (DNA) or RNA-driven = no integrated transgene.	No foreign gene where gene editing nuclease is transiently expressed. Risk of spread of transgene or modified organism. Changes to native genes.	NO	YES [11,12]
Gene edit: Ribonucleoprotein (RNP)-driven change	Precise mutation to native genes only (some of target mutagenesis measurable but minimal).	No foreign gene. Risk of spread of modified organism. More precise genetic changes than chemical, radiation, or UV mutagenesis.	NO	YES [11,12]
